# Electroencephalographic read-outs of the modulation of cortical network activity by deep brain stimulation

**DOI:** 10.1186/s42234-018-0003-x

**Published:** 2018-03-15

**Authors:** Astrid Kibleur, Olivier David

**Affiliations:** 1 University Grenoble Alpes, Grenoble Institut des Neurosciences, GIN, 38000 Grenoble, France; 2 Inserm, U1216, 38000 Grenoble, France; 3 Grenoble Institut des Neurosciences – Chemin Fortuné Ferrini – Bât EJ Safra, 38700 La Tronche, France

**Keywords:** Deep brain stimulation, Electroencephalography, Cortical networks, Signal processing, Functional neuroanatomy

## Abstract

Deep brain stimulation (DBS), a reversible and adjustable treatment for neurological and psychiatric refractory disorders, consists in delivering electrical currents to neuronal populations located in subcortical structures. The targets of DBS are spatially restricted, but connect to many parts of the brain, including the cortex, which might explain the observed clinical benefits in terms of symptomatology. The DBS mechanisms of action at a large scale are however poorly understood, which has motivated several groups to recently conduct many research programs to monitor cortical responses to DBS. Here we review the knowledge gathered from the use of electroencephalography (EEG) in patients treated by DBS. We first focus on the methodology to record and process EEG signals concurrently to DBS. In the second part of the review, we address the clinical and scientific benefits brought by EEG/DBS studies so far.

## Background

Deep brain stimulation (DBS) is used in routine in Parkinson’s disease (PD) (Benabid et al. 2009) and in an extending number of pathologies such as epilepsy, obsessive compulsive disorders (OCD) and treatment resistant depression (TRD) (Perlmutter and Mink 2006). The mechanisms of action of DBS remain debated. Besides local effects that directly modulate the activity of the DBS target (Dostrovsky and Lozano 2002), DBS has widespread effects on the cortex as well by means of activation of afferent and efferent axons, and of fibers passing by the target (McIntyre and Hahn 2010). These cortical modulations remain still not well defined. Some groups have developed electroencephalographic (EEG) methods to address this issue. Here we review the existing literature (Table 1), which indicates that EEG is a valuable tool to gather knowledge on how DBS works on the cortex at a large scale.

**Table 1:** Summary of EEG DBS studies and their principal results. Subjects number were given only for those who had direct stimulation or ON/OFF DBS protocol

Study	Subjects	DBS target	Protocol	DBS effect
Direct effect				
Ashby et al. (2001)	6 PD	STN		CEPs at 3, 5 and 8 ms
Baker et al. (2002)	10 PD, 4 Epi	STN		CEPs from 1 to 400 ms
MacKinnon et al. (2005)	11 PD	STN	Electrical skin stimulation	CEP at 23 ms
Zumsteg (2006)	9 Epi	Thal		CEPs between 20 and 320 ms
Zumsteg (2006)	6 Epi	Thal		CEPs at 24, 34 and 70 ms
Tisch et al. (2008)	6 Pri Gen Dyst	GP		CEP at 26.6 ms
Eusebio et al. (2009)	16 PD	STN		CEP at 21 ms
Walker (2012a)	5 PD	STN		CEPs at 1, 5.7 and 22.2 ms
Walker (2012b)	5 Ess Trem	Thal		CEPs at 0.9, 5.6 and 13.9 ms
ON/OFF designs				
Gerschlager et al. (1999)	10 PD, 10 HC	STN	Go/NoGo	Increased contingent negative variation amplitude
Pierantozzi et al. (1999)	6 PD	STN, GPi	Electrical skin stimulation	Increased frontal N30 amplitude
Gerschlager et al. (2001)	8 PD	STN	Auditory oddball task	No effect on P300 latency
Priori et al. (2001)	9 PD	STN	Electrical skin stimulation	Reduced N20 amplitude
			Passive visual task	Reduced P100 amplitude
Devos et al. (2002)	6 PD	GPi	Wrist flexion movement	Increased contralateral premovement/movement ERD
Devos et al. (2003)	10 PD	STN	Wrist flexion movement	Increased beta ERS
Devos et al. (2004)	10 PD, 10 HC	STN	Wrist flexion movement	Reduced ERD spread and increased ERD amplitude
Insola et al. (2005)	1 PD	STN	Electrical skin stimulation	Increased N20 and N30 amplitudes
Silberstein et al. (2005)	16 PD	STN	Resting state	Reduced beta cortical coupling
Jech et al. (2006)	11 PD	STN	Resting state	Reduced alpha power
			Passive visual task	Reduced N70/P100
Kovacs et al. (2008)	23 PD, 11 HC	STN	Auditory oddball task	P300 amplitude correlated to DBS voltage
Conte et al. (2010)	13 PD, 13 HC	STN	Somatosensory temporal discrimination task	Reduced parietal SEP amplitude
Klostermann et al. (2010)	10 PD	STN	Choice response task	Reduced lateralized readiness potentials
			Oddball task	
Cavanagh et al. (2011)	14 PD	STN	Decision task	Inversed theta power relation to RT
Swann et al. (2011)	15 PD, 15 HC	STN	Stop signal task	Increased right frontal beta power
Broadway et al. (2012)	12 TRD	SCC	Resting state	Increased frontal theta cordance
Selzler (2013)	10 PD, 20 HC	STN	Working memory task	Reduced N200 amplitude and increased N200 latency
Figee et al. (2013)	13 OCD	NAc	Symptom provocation task	Reduced low frequency ERS
Smolders et al. (2013)	8 OCD	NAc	Resting state	Reduced frontal theta phase stability
Quraan et al. (2014)	12 TRD, 15 HC	SCC	Resting state	Frontal theta and parietal alpha asymmetry dependent on clinical response
Hilimire (2015)	7 TRD	SCC	Emotional self referential task	Reduced P1 and P3 amplitudes
Gulberti et al. (2015a)	12 PD, 12 HC	STN	Rhythmic auditory stimulation	Reduced P1/N1 amplitude
Gulberti et al. (2015b)	12 PD, 12 HC	STN	Rhythmic auditory stimulation Resting state	Normalized beta modulation Reduced beta power
Sun et al. (2015)	20 TRD	SCC	Working memory task	Reduced frontal gamma and beta power and increased theta-gamma coupling
Kibleur (2016)	12 OCD	STN	Stop signal task	Reduced P300 amplitude and increased P300 latency and reduced basal ganglia to right frontal cx connection strength
Kibleur (2017)	5 TRD	SCC	Emotional Stroop task	Reduced N170 amplitude and reduced temporal pole to visual ventral cx connection strength

*PD* Parkinson’s Disease, *Epi* epilepsy, *Pri Gen Dyst* primary generalized dystonia, *Ess Trem* Essential Tremor, *HC* healthy control, *TRD* treatment resistant depression, *OCD* obsessive compulsive disorder, *STN* subthalamic nucleus, *Thal* thalamus, *GP* globus pallidus, *GPi* globus pallidus internus, *NAc* nucleus accumbens, *SCC* subcallosal cingulate cortex, *SCEP* subcortical-cortical evoked potential, *ERS* event related synchronization, *ERD* event related desynchronization, *SEP* somato-sensory evoked potential, *RT* reaction time, *cx* cortex

## Methods for studying cortical responses to DBS with EEG

### DBS artefact

The study of DBS-induced cortical modulation can be performed from different perspectives, either by using cognitive protocols or by studying directly cortical activation following DBS pulses. However, DBS pulses usually induce high amplitude artefacts on EEG recordings, limiting its use.

Depending on the stimulation parameters, the DBS artefact does not need necessarily to be corrected. For instance, with short pulse width or bipolar stimulation, the scalp DBS induced artefact is sharp which enables quantification of fast responses, as early as 3 ms post stimulation (Ashby et al. 2001). This is not the case with monopolar stimulation that induces artefacts up to 30 ms (with DBS of the subthalamic nucleus, STN) (MacKinnon et al. 2005) and 50 ms (with DBS of the globus pallidus internus, GPi) (Tisch et al. 2008) post stimuli, thereby hiding early responses (Zumsteg et al. 2006; Eusebio et al. 2009). Experimentally, it is possible to minimize the presence of the DBS artefact in event-related responses by alternating the anode and cathode electrode contacts, which reverses the sign of the artefact but not that of the neuronal responses. Therefore, averaging recordings with inverted cathode and anode minimizes the artefact amplitude and enables visualization of evoked responses, as early as 1 ms after the artefact (Walker et al. 2012a, b).

**Fig. 1:**
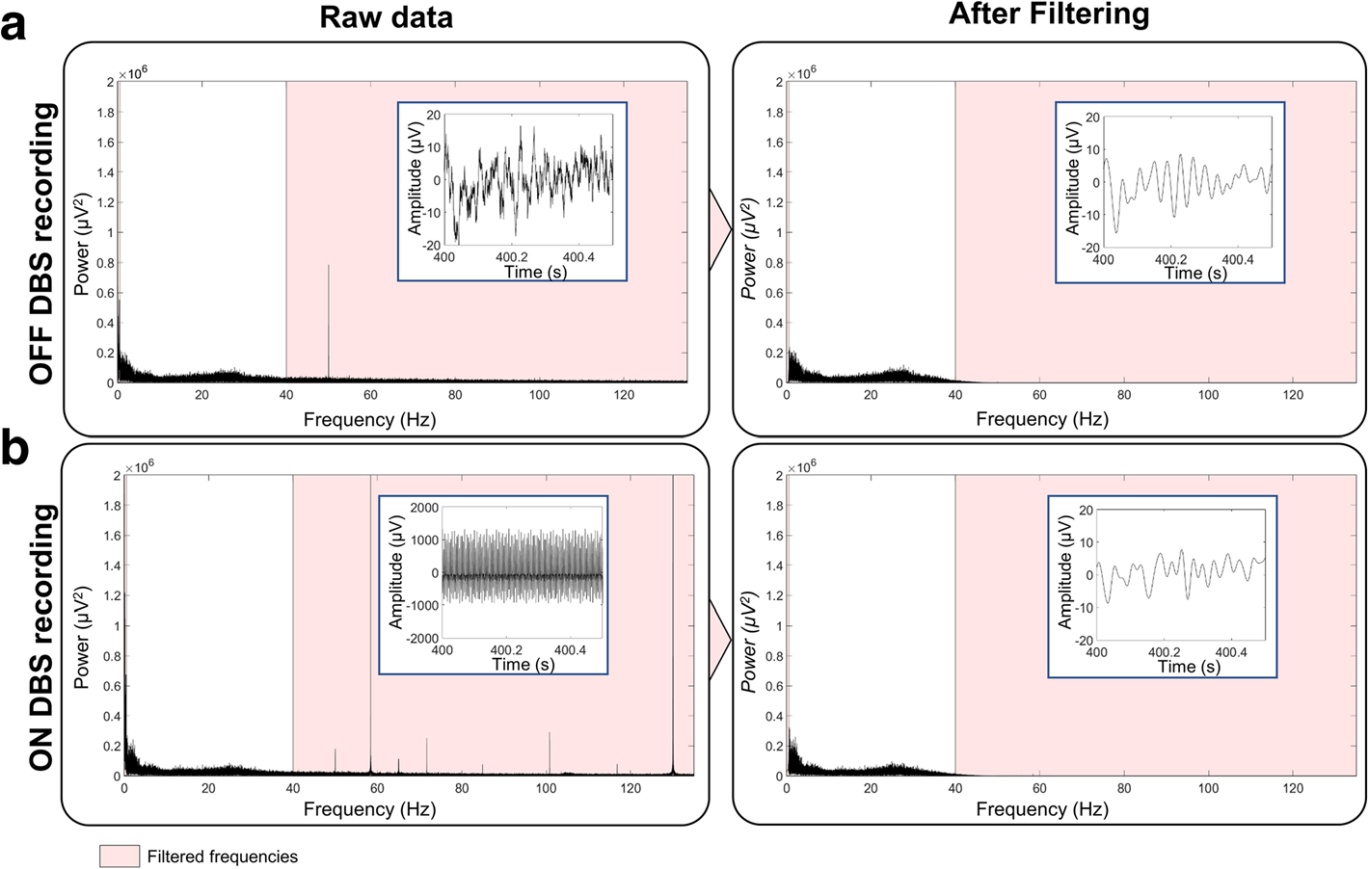
Power spectrum and EEG time series at rest before (left) and after (right) correction of the DBS artefact. **a** With DBS turned OFF. **b** with DBS turned ON. The EEG recording was obtained in a PD patient stimulated at 130 Hz bilaterally in the STN. A low pass filter with a cut-off at 40 Hz was applied to remove the DBS artefact. See (Kibleur et al. 2016) for full description of the data acquisition procedure

Because EEG is best suited to record activity below 40 Hz, in particular for event-related responses, low-pass filtering (e.g. with a 50 Hz cutoff (Cavanagh et al. 2011; Swann et al. 2011; Selzler et al. 2013)) is usually sufficient to remove the DBS artefact and its harmonics when DBS is applied at high frequency, e.g. 130 Hz (Fig. 1). However, using narrow band-pass filters can produce synchronized artefactual activity from ringing artefacts (Yeung et al. 2004). In some cases, there are still high amplitude aliasing artifacts with lower frequencies, which can be corrected individually using notch filters (Jech et al. 2006; Kibleur et al. 2017) and/or matched filter method which consists in modeling the artifact of the recordings with combination of sinusoidal waves (Sun et al. 2014).

If monovariate spectral filtering is insufficient, spatial methods of signal decomposition can be used, e.g. independent component analyses. The components corresponding to the DBS artefact can be identified and removed from the data based on their typical topographical distribution (focal above electrodes) and their temporal and spectral patterns (Gulberti et al. 2015a, b).

### Subcortico-cortical evoked responses

**Fig. 2:**
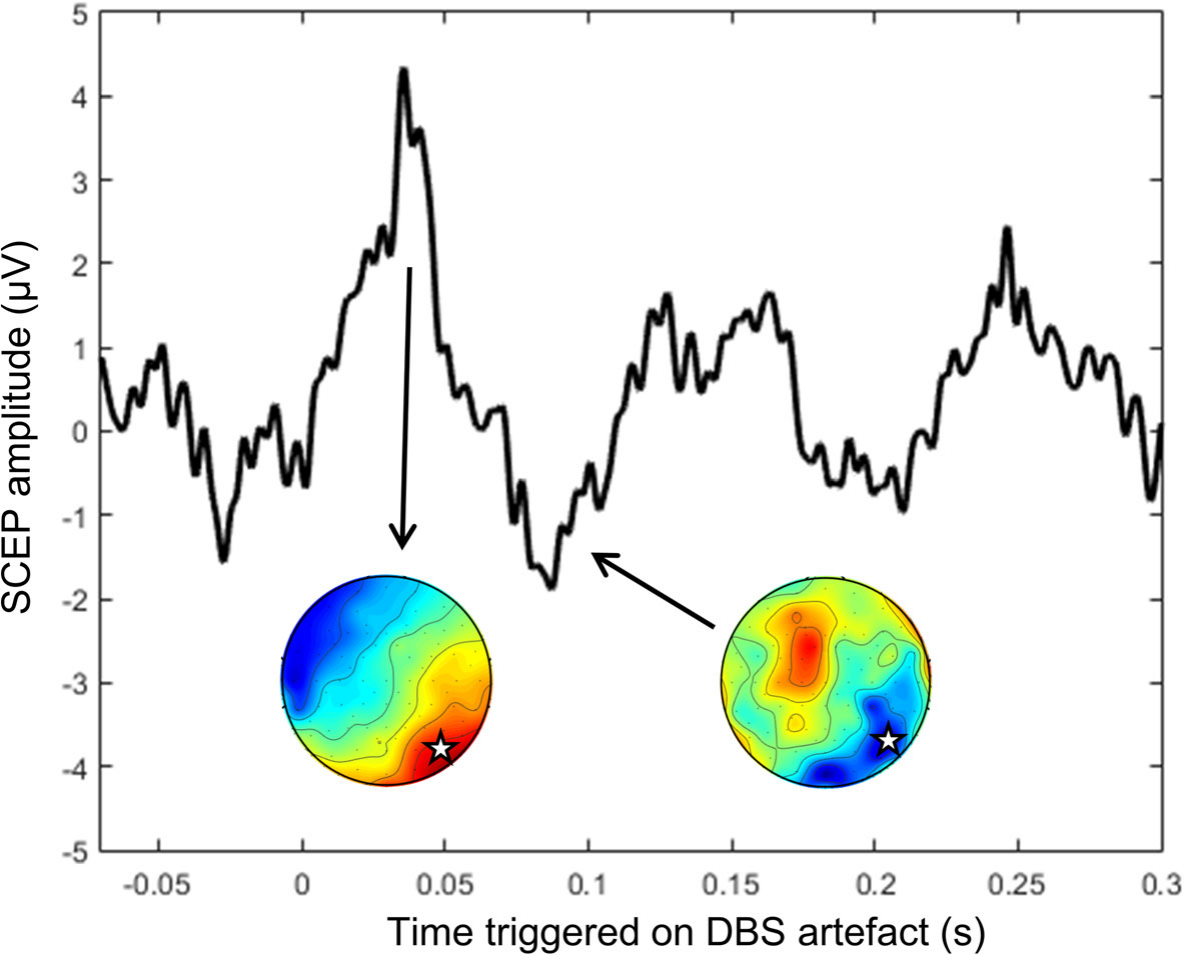
SCEP on a right occipital electrode in a PD patient stimulated in the left STN at 3 Hz. Topographical EEG plots show the two main components of the SCEPs. The white star indicates the position of the electrode used to compute the SCEP. Kibleur et al., unpublished data

The cortical networks modulated by DBS can be studied by recording their electrophysiological responses to single pulses, the so-called subcortico-cortical evoked potentials (SCEPs, Fig. 2) (MacKinnon et al. 2005; Zumsteg et al. 2006; Baker et al. 2002), to paired pulses or to bursts of high frequency stimulation (Baker et al. 2002). SCEPs are built by triggering the stimulation artifact, epoching and averaging over hundreds of events. Then, from the electrode contact position obtained using post-operative MRI images, a cortical mapping of DBS from SCEPs features can be established (Ashby et al. 2001; Tisch et al. 2008). The effect of DBS parameters on SCEPs amplitude and spatial patterns can be studied as a function of DBS frequency (Eusebio et al. 2009) or voltage (Walker et al. 2012a; b). EEG source reconstruction of SCEPs can be used to improve the DBS cortical mapping, for instance with LORETA method (Zumsteg et al. 2006). It has also been proposed to combine SCEPs and somatosensory evoked potentials (SEPs) on the same patients (MacKinnon et al. 2005). It was suggested that SCEPs and SEPs medium latency components may originate from the same cortical regions because of their similar scalp topography.

### Task-related evoked responses

The impact of DBS on cortical networks can be studied during specific tasks (sensory, motor, executive, cognitive or emotional) by comparing EEG recordings with DBS turned ON or OFF, in a sequential manner (Smolders et al. 2013), in randomized counterbalanced order (Cavanagh et al. 2011; Swann et al. 2011; Selzler et al. 2013) and double blind fashion (Kovacs et al. 2008). Both EEG sessions can be recorded on the same day consecutively without electrode repositioning between sessions (Devos et al. 2004).

Alternating EEG recordings with DBS ON and OFF requires to be careful about the DBS washout effect: when turning the stimulator OFF, there might be still ongoing DBS ON carry over effects (Gulberti et al. 2015b), such as the modulation of synaptic plasticity induced by chronic DBS that is not washed out by brief DBS discontinuation (Gulberti et al. 2015a; Quraan et al. 2014). Therefore, it is important to wait sufficiently long, given the ethically acceptable conditions that depend on the pathology, between the DBS setting modification and the beginning of task-related behavioral and EEG recordings. The wash-out time required is highly dependent on the associated symptoms, target and pathology, and hence on the studied brain networks. For instance, in PD, motor symptoms are very quick (a few minutes (Moro et al. 2002)) to appear when the stimulation is turned OFF whereas in TRD, the depressive symptoms may take much more time to come back (from few hours up to several weeks (Mayberg et al. 2005)).

Chronic DBS effects on cortical networks can also be studied with DBS discontinued just before the EEG recording in order to avoid any effect of the DBS artefact in the data analysis. This has been used in several longitudinal studies to look at the long-term plastic effects of DBS, with EEG recordings before DBS implantation and then at several time points during chronic DBS treatment (Broadway et al. 2012; Hilimire et al. 2015). A similar procedure has also been used to contrast a condition where DBS was switched OFF for 12 h (OFF state) and a condition where DBS was switched OFF just before the recording (ON state) after a long ON DBS period (Pierantozzi et al. 1999), assuming that the DBS post-effect period can last up to 3 h (Devos et al. 2002). These methods bypass the stimulation artifact issue but they remove the acute DBS effects on the brain. Indeed, for instance, the effect of DBS on SEPs faded away progressively in 1 h after switching the DBS OFF (Pierantozzi et al. 1999). Therefore, this method may lead to underestimations of DBS effects.

**Fig. 3:**
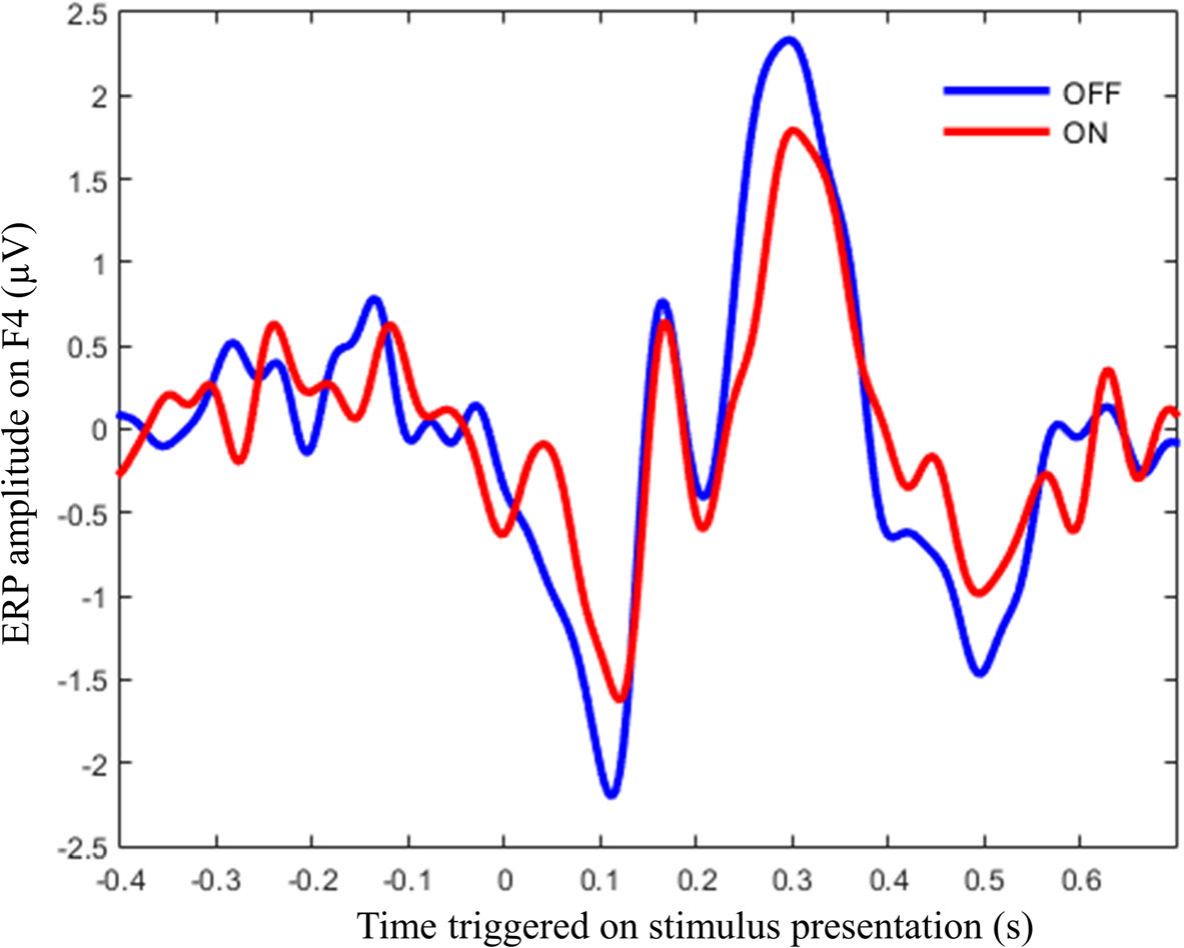
Modulation of a cognitive ERP by DBS. This plot shows a grand average over 12 OCD patients stimulated in the STN at 130 Hz of the ERP in right fronto-central electrodes during a stop signal task. The amplitude of the ERP was reduced when the stimulation was ON (red) vs. when it was OFF (blue). See (Kibleur et al. 2016) for full description of the data acquisition procedure

Acute DBS effects can be studied through the modulation of task event-related potentials (ERPs), in terms of amplitude, shape and latency, by switching DBS ON and OFF (Fig. 3). In TRD patients, this approach was used to study the effect of subgenual cingulate cortex (SCC) stimulation during an emotional word recognition task (Hilimire et al. 2015) and an emotional Stroop task (Kibleur et al. 2017). In OCD patients, it was used to study the role of the associative-limbic STN on the cortical networks of motor inhibition during a stop signal task (Kibleur et al. 2016). The same methodology was also used to study ERPs amplitude and latency modulation by DBS on visual evoked potentials (Jech et al. 2006; Priori et al. 2001), in a passive rhythmic auditory stimulation task (Gulberti et al. 2015b), in a working memory task (Selzler et al. 2013) and in an auditory Go/NoGo task in PD patients stimulated in the STN (Gerschlager et al. 1999; 2001) and on SEPs in PD patients with STN (Priori et al. 2001; Conte et al. 2010; Insola et al. 2005) and GPi DBS (Pierantozzi et al. 1999). As for SCEPs, the reconstruction of ERP sources, for example using minimum norm (Kibleur et al. 2016) or multiple sparse priors (Kibleur et al. 2017), can help defining the projection of DBS modulation on cortical regions activated by the specific cognitive tasks. In addition, dynamical causal modelling can also address the issue of how DBS modulates subcortico-cortical and cortico-cortical effective connectivity (Kibleur et al. 2016, 2017).

Beyond ERPs, task-related DBS effects can also be described from macroscopic neural oscillations using spectral analyses time-locked to stimulus presentation. Averaging across trials then gives a time frequency representation of the evoked activity. EEG power modulation by DBS was studied in PD patients (stimulated in the STN and/or GPi) on evoked beta power in an inhibition task (Swann et al. 2011), on alpha desynchronization and beta synchronization in a motor task (Devos and Defebvre 2006) and a passive rhythmic auditory stimulation task (Gulberti et al. 2015a). Time frequency maps were also computed in a working memory task in STN DBS PD patients (Selzler et al. 2013), in STN DBS OCD patients during a symptom provocative task (Figee et al. 2013) and in SCC DBS TRD patients (Sun et al. 2015). Then, by focusing on frequency bands of interest, the DBS modulation of the spectral power or cordance (sum of normalized absolute and relative theta power) can be studied in terms of amplitude and peak latency, as in PD patients in a motor task (Devos et al. 2004) or in TRD patients at rest (Broadway et al. 2012).

To study non-phase locked (induced) responses, a trial to trial analysis must be used, for instance, to show the DBS modulation of theta power regression with response time in a decision task (Cavanagh et al. 2011). The DBS effects on coherence spectrum (Silberstein et al. 2005), on phase coherence (Quraan et al. 2014) or on phase amplitude coupling (Sun et al. 2015) were also studied to assess differences in cortico-cortical coupling. To study neural communication between brain nodes, the resting state phase preservation index (quantifying the phase stability of an oscillation) was proposed as a potential tool of interest for quantifying the frontal theta activity in STN DBS OCD patients (Smolders et al. 2013).

## Advances in therapy and neurophysiology from DBS-EEG studies

EEG thus offers many ways to study the mechanisms of action of DBS at the cortical level, which should enable to improve DBS methodology for optimal clinical outcomes and to increase our understanding of human neurophysiology.

### Improving parameters setting for DBS therapy

Improving DBS efficacy is an important clinical research objective. Conclusions from EEG-DBS studies focused on the mapping of subcortico-cortical projections may enable to refine DBS targeting. For instance, in the STN, more ventral stimulation elicited stronger SCEP at 3 ms (Ashby et al. 2001), and more dorsal stimulation increased the amplitude of the medium latency of SCEP (peak around 23 ms) (MacKinnon et al. 2005). In pallidal DBS in primary generalized dystonia, contacts located more ventrally (corresponding to the clinically effective contacts) elicited larger medium latency SCEP (Tisch et al. 2008). However, even if cortical activation was tightly dependent on electrode position (Zumsteg et al. 2006), direct cortical activation could produce relatively similar evoked responses with different targets (such as the anterior and dorsomedial nuclei of the thalamus (Zumsteg et al. 2006)). Studies of SCEPs also showed some links between DBS parameters and cortical response patterns that could be related to clinical response. For example, monopolar DBS of the thalamus induced cortical responses four times higher in amplitude than bipolar DBS (Zumsteg et al. 2006). This amplitude modulation was highly dependent on electrode’s impedance. Early SCEP amplitude and frequency were related to clinical effect in tremor patients stimulated in the thalamus, which suggests that this SCEP component could be used to choose DBS parameters optimally (Walker et al. 2012a).

In studies correlating the clinical evolution with the EEG modulation by DBS, predictive biomarkers of treatment response could be outlined. Theses markers can in principle be assessed before implantation and thus bring information for surgical decision. For instance, in TRD where SCC DBS efficacy was shown to be highly heterogeneous across patients, low frontal theta cordance predicted greater clinical improvement after 24 weeks (Broadway et al. 2012). Because of its sensitivity to DBS parameters, EEG modulation by DBS can theoretically be used to help optimizing DBS parameters to reach best clinical outcome with least side effects. In PD patients, STN DBS induced a decrease of early visual evoked potentials which was proportional to the intensity of power increase (Jech et al. 2006) and the fronto-central P300 amplitude from an oddball auditory task was correlated with the stimulation voltage (Kovacs et al. 2008).

EEG biomarkers are particularly useful in pathologies where DBS effects on symptoms are not immediately observed, such as in psychiatric diseases. For instance, in TRD patients, frontal theta cordance increase at 4 weeks predicted stronger clinical benefit at 24 weeks (Broadway et al. 2012). Furthermore, the clinical efficacy of subgenual cingulate DBS in those patients was correlated with decreased right frontal gamma oscillations and increased left frontal theta-gamma coupling during a working memory task (Sun et al. 2015). In OCD patients, nucleus accumbens (NAc) DBS was suggested to improve the symptoms by inducing a reduction of frontal theta phase stability (Smolders et al. 2013). Measuring this index for several stimulation parameters could thus be a way to find optimal DBS parameters.

EEG DBS studies can also validate the use of DBS by comparing its effects on EEG markers with the effects of best medical treatments, or by comparing these effects with the same markers in healthy control groups. For instance, it has been shown that STN DBS in PD patients normalized the movement related desynchronization (Devos et al. 2004), the post-movement beta synchronization (Devos et al. 2003), the central beta cortico-cortical coupling at rest (Silberstein et al. 2005) and the beta modulation evoked by fast rhythmic auditory stimulation (fRAS) (Gulberti et al. 2015a) to near normal patterns and that these effects were similar to the ones induced by L-dopa treatment. Using fRAS, another study showed that the normalization of early ERP amplitudes was specific to STN DBS action and that dopaminergic treatment did not restore a normal pattern (Gulberti et al. 2015b). It was also shown in PD patients that STN and GPi DBS induced an increase of the SEPs (Pierantozzi et al. 1999) and partially restored movement-related spectral patterns similarly to dopaminergic drugs (Devos and Defebvre 2006). Moreover, STN DBS in PD patients normalized (compared to healthy subjects) the working memory N200 amplitude and latency (Selzler et al. 2013), the lateralized readiness potentials latency in a choice response task (Klostermann et al. 2010) and the fronto-central contingent negative variation amplitude (pre-stimulus negative potential shift) but did not change the P300 latency, which was shortened by levodopa treatment, in a Go/NoGo task (Gerschlager et al. 1999, 2001).

Finally, EEG studies of DBS cortical effects may be used to investigate stimulation side effects mechanisms. For instance, DBS induced increase of impulsive behavior in high conflict decision was shown to be related to decreased interactions between STN and mesial prefrontal cortex (Cavanagh et al. 2011). The functional connectivity between these two structures might thus be a good target in order to reduce impulsivity related to DBS therapy.

### Understanding better functional neuroanatomy

EEG studies can be used to investigate the differential effects of DBS on brain dynamics. In longitudinal studies, modulation of different processes may require different DBS durations. This was shown in TRD patients in a self-referential task where SCC DBS induced after 1 month a reduction of the automatic processing of negative information (as shown by an effect on the early ERPs) and after 6 months a reduction of the controlled processing of this information (as shown by an effect on later ERPs) (Hilimire et al. 2015).

EEG-DBS studies can also improve our understanding of DBS mechanisms of action on brain networks by measuring the remote neurophysiological effects of DBS on various cortical regions. For example, STN DBS in PD was shown to increase cortical beta activity in a motor inhibition task, suggesting a DBS-induced improvement of information transfer from the basal ganglia to the cortex (Swann et al. 2011). The modulation of brain networks with DBS can also be used to synchronize DBS target activity at specific frequencies. By studying the modulation of SCEPs by dopamine in PD patients, STN-cortical networks were shown to resonate at around 20 Hz, depending on dopamine intake which could limit the induced amplitude increase at this frequency (Eusebio et al. 2009).

Assuming that early SCEPs are generated by cortical regions directly connected to the DBS target, inferences on the nature of the conducting elements can be made according to SCEP latencies. For instance, the SCEP occurring before 8 ms from STN stimulation were proposed to originate from antidromic activation of premotor and motor cortex connections to the STN (Ashby et al. 2001). Furthermore, the SCEP observed at 3 ms could be evoked with low stimulation power implying that it could originate from the activation of myelinated axons, which have low activation threshold (Ashby et al. 2001). This early SCEP component could be equivalent to the 1 ms latency component found in another study that was hypothesized to originate from non-synaptic antidromic activation (Walker et al. 2012b) due to its short latency and refractory period. The frontal early SCEP might be related to STN DBS clinical efficacy in PD whereas later SCEPs (after 20 ms), which represent indirect (polysynaptic) cortical activation, may implicate networks not strongly involved in the clinical improvement (MacKinnon et al. 2005).

The modulation of effective connectivity by DBS, either at the subcortico-cortical or at the cortico-cortical levels, can also be studied from EEG signals. In TRD patients responders to subgenual cingulate DBS, DBS was shown to normalize (compared to control subjects) resting state alpha and theta power asymmetry and long range functional connectivity between left fronto-central and right parietal regions (Quraan et al. 2014). In TRD patients, effective connectivity from the temporal pole to the fusiform gyrus was decreased with SCC DBS (Kibleur et al. 2017). In OCD patients, it was shown that subcortico-cortical effective connectivity was the most modulated connection by STN DBS in a motor inhibition task (Kibleur et al. 2016).

## Conclusion

EEG-DBS methodology is an interesting approach to better understand the functional neuroanatomy of the human brain. EEG is safe and cheap and can be easily conducted in many clinical neurophysiology environments. It is appropriate for DBS studies but also for other kinds of electrical stimulation, such as vagus nerve stimulation (Corazzol et al. 2017; Clarençon et al. 2014; Kibleur n.d.). Furthermore, new advances in closed-loop DBS (Osorio et al. 2001; Broccard et al. 2014; Parastarfeizabadi and Kouzani 2017) aim at optimizing stimulation parameters using neuronal and/or physiological feedbacks to obtain the best effects with the lowest electrical consumption. The beneficial use of scalp EEG for closed-loop DBS still needs to be demonstrated.

It is thus important to keep continuing characterizing better the DBS footprints on cortical activity as recorded by EEG, even though EEG spatial resolution will remain intrinsically limited to few centimeters. Unfortunately, the post-processing of EEG data is complex and time-consuming. Important issues remain to be addressed, such as improving noise correction and testing the stability and repeatability of EEG markers of DBS mechanisms of action. In the future, it will be important to better homogenize the way the data are recorded and processed by using shared methods in open-source processing toolboxes. Another aspect to standardize such studies would be to better control important factors such as medication, wash-out duration and stimulation parameters. Finally, data sharing between international DBS centers is a meaningful way to quickly improve the statistical validity of the main findings.
